# RegionSizeR– A Novel App for Regional Sample Size Planning in MRCTs

**DOI:** 10.1007/s43441-024-00679-6

**Published:** 2024-08-08

**Authors:** Guannan Sun, Xin Sun, Huijuan Su, Yuqin Liao, Di Wei, Hanqing Ma, Xinyu Li, Ran Fan, Xiaowei Ren

**Affiliations:** 1 Pharmaceuticals Statistics and Data Insights, Bayer Healthcare Co. Ltd. Research & Development, 6F Parkview Green Blk B, No.9 Dongdaqiao Road, Beijing, China; 2 Bayer US LLC, Whippany, NJ USA; 3 Akesobio Inc, Nanjing, China

**Keywords:** Sample size, Regions joining MRCT, Preservation of treatment effect, Consistency probability, Clinical trial

## Abstract

In multi-regional clinical trials, planning the sample size for participating regions is essential for the evaluation of the treatment effect consistency across regions. Based on the MRCT design and sample size allocation to regions, consistency probability is usually used to predict the consistent trend between regions and the overall population, while preserving a certain proportion of the overall treatment effect. Specific enrollment characteristics in a region of interest should also be considered during the time of the sample size planning. To facilitate efficient and harmonized regional sample size planning, we have developed *RegionSizeR*, a comprehensive and user-friendly interactive web-based R shiny application that can be obtained from https://github.com/rsr-ss/RegionSizeR. This simulation-based app can serve as an initial point for discussions on sample size allocation plans, following preservation of treatment effect method in ICH E17. The app accommodates various types of endpoints and designs, including continuous, binary, and time-to-event endpoints, for superiority, non-inferiority, and MCP-Mod designs. To ensure the validity of this app, independent testing is conducted allowing a discrepancy of no more than 1% across all results considering various scenarios.

**Supplementary Information:**

The online version contains supplementary material available at 10.1007/s43441-024-00679-6.

## Introduction

In the context of a Multi-Regional Clinical Trial (MRCT), the key consideration for planning regional sample size is to ensure the meaningful evaluation of consistency across regions and to provide the necessary information to support regulatory decision making. There are several strategies for regional allocation that have been suggested according to ICH E17. In terms of practical implementation, strategies for regional sample size allocation should balance between scientific objectives and logistical considerations [1].

The *Biostatistics Innovation Center (BIC)* at Bayer AG is composed of self-organized working groups which collaborate on statistically relevant topics of their interests [2, 3]. One such working group within *BIC* is dedicated to statistical considerations involved in joining MRCTs. The initial objectives of this BIC working group, named ‘Bridging Strategies and Consistency Assessments on Clinical Outcomes from Asia Perspectives’, were to provide a brief review of regulatory requirements in Asian countries and to develop programming tools for planning reginal sample size in MRCTs, among other goals. As an offshoot of this working group’s effort, an efficient and harmonized package for planning regional sample sizes in MRCTs– named *RegionSizeR*, is developed using R [4] and implemented via the web-based R shiny application [5].

In this paper, instead of individual users creating their own programming codes, the *RegionSizeR* app which extends the concept designed by *BIC* working group is introduced. Section “Methods and Illustrations” explains the comprehensive features of the app followed by detailed illustrations of how it works using hypothetical parameter settings for different scenarios. Section “Discussion and Conclusion” concludes the paper and discusses more context of the app concerning developing and validation.

## Methods and Illustrations

**Fig. 1:**
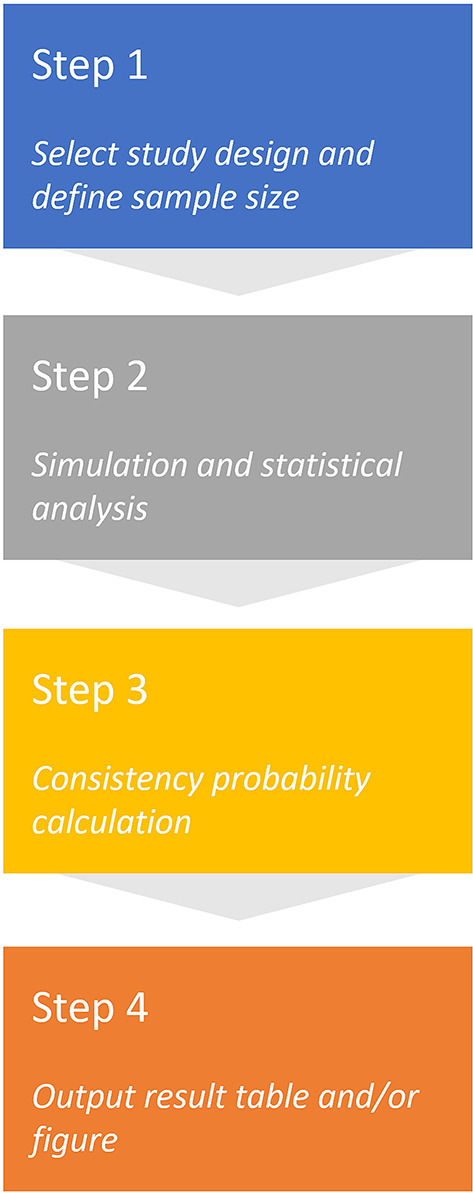
Working flow of the app with 4 steps

For regional sample size planning in *RegionSizeR*, there are mainly 4 steps (see Fig. 1). After receiving global and regional parameters entered in Step 1, the overall outcomes for the chosen type of endpoint are simulated using R and participants from a certain region are randomly sampled in Step 2. Step 2 is usually replicated for thousands of times for more robust outputs. Based on the estimation of the simulated treatment effects in the overall population and subpopulation corresponding to the region of interest, the consistency probability is then calculated preserving a pre-specified proportion of the overall treatment effect (π) in Step 3. The result table containing conditional (given statistically significant of the overall population) and unconditional (regardless of the overall population result) consistency probabilities together with key input parameters can be viewed and downloaded in Step 4. More details on the consistency probability definitions can be found in Sect. “Superority Design with Continuous and Binary Endpoint”, “Non-Inferiority Design”, and “MCP-Mod Design”. In addition, the scatter plot of consistency probability vs. subpopulation proportion is provided for superiority and non-inferiority (NI) designs.

The *RegionSizeR* app considers three different types of design that are typically used in late phase development to evaluate regional treatment effect consistent trend:

- Superiority design;
- NI design;
- Multiple Comparisons Procedure– Modelling (MCP-Mod) design.

**Fig. 2:**
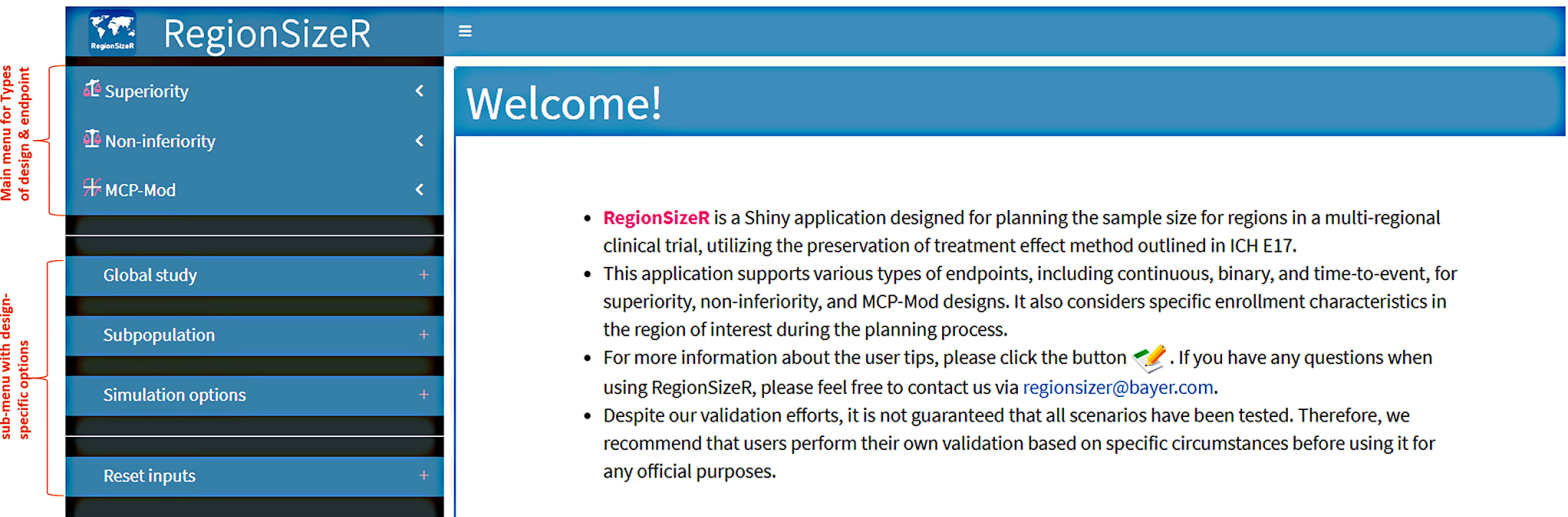
Screenshot of control panels (main menu and submenu) with the Welcome page of the app. The main menu contains the options for different types of trial designs and endpoints; while the submenu includes the parameters specific to the chosen endpoint based on overall and subpopulation information. The Welcome page describes the app briefly and provides a link to the user tips in details together with the contact information of the supporting team

For each type of study design, three types of endpoints including continuous, binary, and time-to-event (TTE, not included in the MCP-Mod design) endpoints can be simulated with various assumptions. Study designs and parameters common to each type of endpoint can be accessed in the main menu on the upper left side of the app (see Fig. 2). After selecting designs and endpoints in the main menu, a sub-menu with design specific options appears on the lower left side, as well as endpoint specific options on the right side of the app. Upon all input information is available, the app checks the parameter values before running the program and user may download/upload this set of inputs to local drive for future use or update.

The global parameters, including total sample size, overall randomization ratio, significant level, and assumed treatment effects etc., are the ones used for study sample size determination provided in the study protocol; While the regional/subpopulation parameters, such as the participant proportion in the subpopulation, randomization ratio in the region, and regional accrual pattern (only for TTE endpoint), leave opportunities to the users taking into account of region-specific considerations. In addition, three modes of joining strategy which are region ‘completely included’, ‘partially included’ with certain including proportions, or ‘completely excluded’ in/from the global study, can be assumed for the simulation depending on regional timeline hurdles or feasibility limitations.

In this paper, we use hypothetical parameters to illustrate the key functions of the app, which is validated by independent programming without controlling of the simulation seeds, based on pre-specified validation plans for different scenarios with an approximately 1% discrepancy allowance. Table 1 in the supplementary materials shows an example of the validation plan used for binary endpoint in superiority design. Similar plans are specified for other types of endpoints or designs adjusting for additional needs of the parameters.

### Superority Design with Continuous and Binary Endpoint

For continuous (and binary) endpoints in superiority designs, denoting $\:{\widehat{\theta\:}}_{R}$ as the regional estimated treatment effect and $\:{\widehat{\theta\:}}_{All}$ as the overall estimated treatment effect based on the simulated data, the consistency probability can be calculated as [6, 7]:

- Unconditional: $$\:prob({\widehat{\theta\:}}_{R}>\pi\:{\widehat{\theta\:}}_{All})$$;

- Conditional: $$\:prob\left(\begin{array}{l}{\widehat{\theta\:}}_{R}>\pi\:{\widehat{\theta\:}}_{All}|{\widehat{\theta\:}}_{All}\:is\\\:statistically\:significant\end{array}\right)$$.

#### Continuous Endpoint with T Test Illustration

**Fig. 3:**
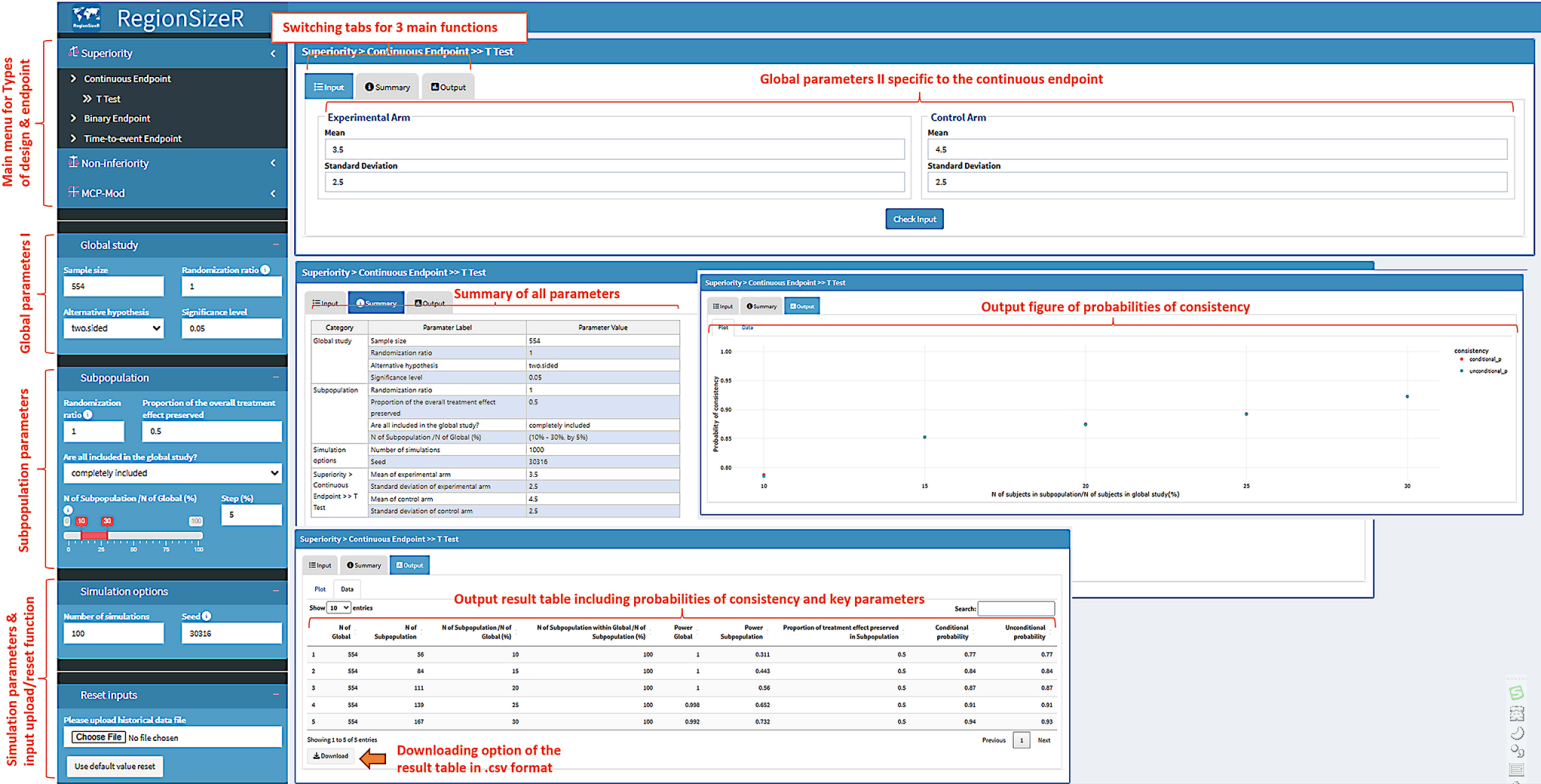
Screenshot of the parameters and switching tabs based on continuous endpoint in superiority trial. The overall and subpopulation parameters are illustrated using a hypothetical example. The switching tabs contains 3 functions that are input (for further parameters), summary (of all input), and output (including a figure and a result table)

As shown in Fig. 3, T test under Superiority Continuous Endpoint is selected in the Main menu, so that global parameters part I (in the sub-menu) and part II (on the right side) used for study sample size determination are available to users to input. In this example, the overall sample size is 554 with 1:1 allocation ratio between two arms and 0.05 type I error rate of the two-sided test. The Mean response of the experimental arm is assumed to be 3.5 unit with standard deviation of 2.5; while the control mean is assumed as 4.5 unit with the same standard deviation as for the experimental arm.

In addition to the global parameters, the panel for subpopulation parameters under the one for global parameters part I is open to users. In this illustration, the treatment allocation in the subpopulation is maintained as 1:1 and all participants in the subpopulation are completely included the overall population as of 10–30% percent with 5% increment (i.e., the subpopulation sizes of 56, 84, 111, 139, and 167 are under considerations for regional sample size planning). For rare cases when only partial/none of participants in the subpopulation can be included in the overall population due to enrollment or timeline hurdles, users can choose one of the other two options (‘partially included’ with certain including proportions or ‘completely excluded’) for the question of ‘Are all included in the global study?’ within this panel.

After setting up the number of simulations and seed (as needed) in the simulation options penal, the ‘check input’ button shall be clicked so that all input parameters are summarized in the second switching tab next to the ‘input’ tab. Besides simple summary, the app also performs logic checks based on the natural relationship among all parameters to make sure the simulation can be conducted smoothly in the next step. For better efficiency, users can download the summary parameters for future use by clicking ‘download’ button. The historical downloads can be uploaded from local drive using the bottom left panel and directly move to the ‘check input’ step.

When the input parameters are all set, the ‘Run’ button finally triggers the simulation and the ‘output’ tab shows next to the ‘summary’ tab when the calculation of consistency probabilities is finished. The output includes both a plot of the conditional/unconditional probability across the range of subpopulation percentage and a result table including probabilities of consistency and key parameters used for the simulation. The Power of the study is provided in the result table to cross check with overall sample size determination assumptions. The result table can be downloaded in.csv format for documentation or further comparison of scenarios as needed.

#### Binary Endpoint with Chi-Square Test Illustration

**Fig. 4:**
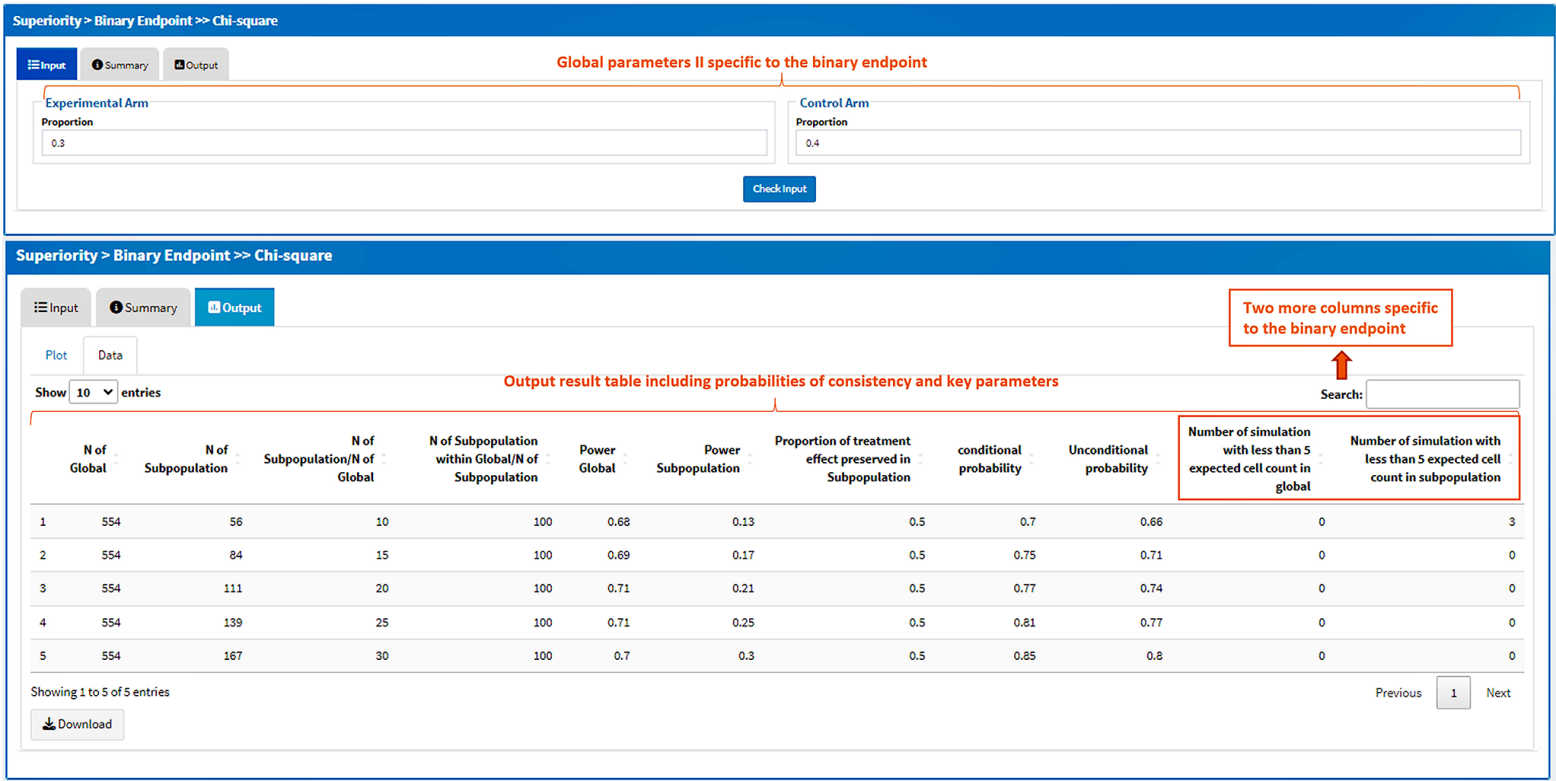
Screenshot of the parameters and result table specific to the binary endpoint. Two more columns are provided in the result table for binary endpoint

For binary endpoints using the Chi-square test, the global parameters (part II) include the proportions of response of both treatment arms (Fig. 4) with the usage of all the other parameters remaining unchanged. In the result table of the output, there are two more columns providing the numbers of simulations with < 5 expected cell count in overall and subpopulation, in case the Fisher’s exact test is more appropriate than the Chi-square test and the probabilities shall be interpreted with cautious.

### Superority Design with TTE Endpoint

For TTE endpoint in superiority designs, denoting $\:{\widehat{HR}}_{R}$ as the regional estimated hazard ratio and $\:{\widehat{HR}}_{All}$ as the overall estimated hazard ratio based on the simulated data, there are two ways of consistency calculation [6, 7, and 11]:

1. Log HR: $$\:\frac{\text{l}\text{o}\text{g}\left({\widehat{HR}}_{R}\right)}{\text{l}\text{o}\text{g}\left({\widehat{HR}}_{All}\right)}>\pi\:$$

(2) Risk reduction: $$\:\frac{1-{\widehat{HR}}_{R}}{1-{\widehat{HR}}_{All}}>\pi\:$$

#### TTE Endpoint with Log-Rank Test Illustration

**Fig. 5:**
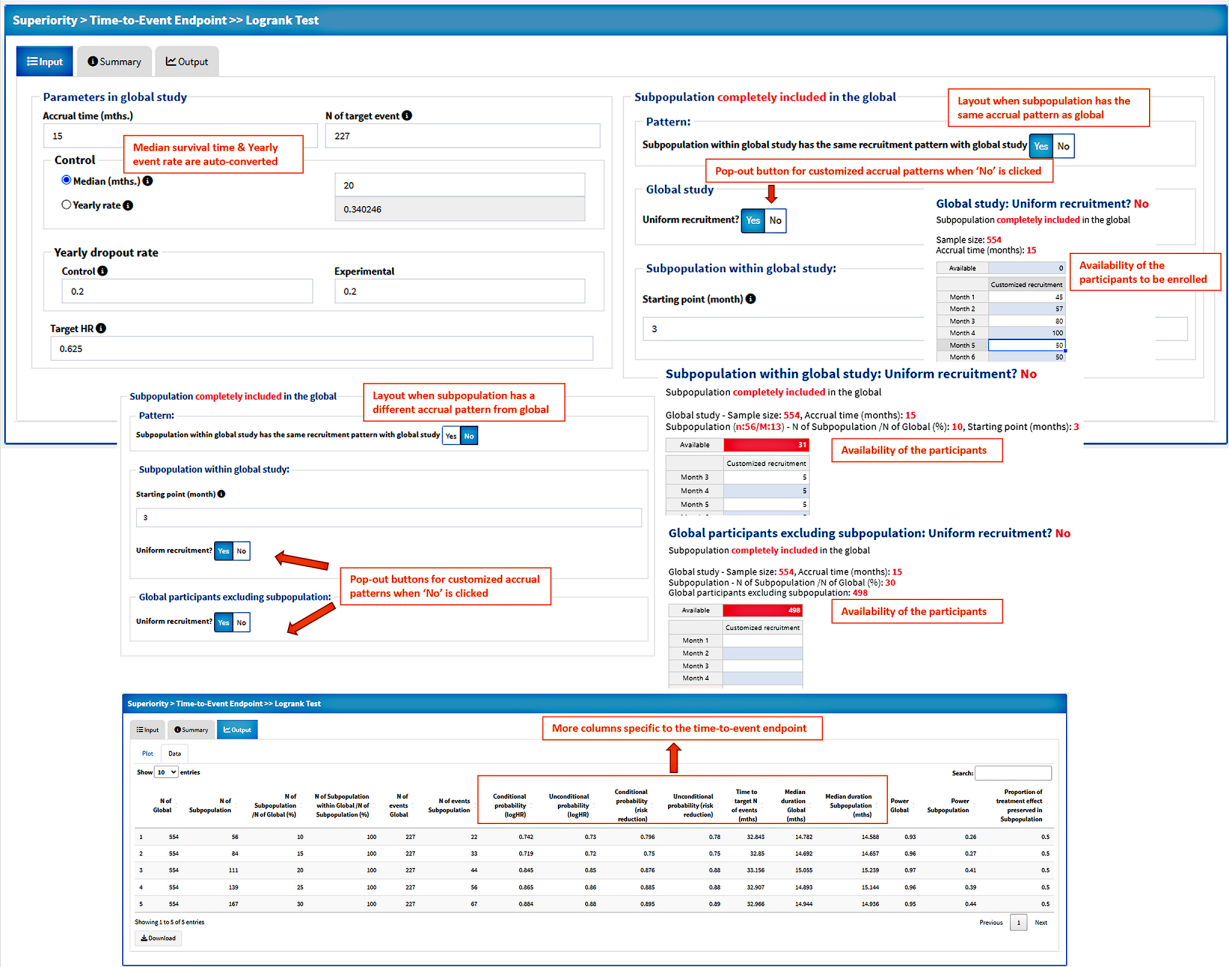
Screenshot of the parameters and result table specific to the TTE endpoint. Specific parameters related to enrollment pattern in overall and subpopulation are illustrated for TTE endpoint, especially when the accrual is different in the region from overall or it is not uniform. More columns specific to TTE endpoint are provided in the result table

For TTE endpoint using the log-rank test, the global parameters (part II) and further subpopulation parameters in the right panel include more complicated options to cover customized enrollment pattern as shown in Fig. 5. The median survival time and the yearly event rate of the control arm in the global study can be converted automatically using this app. The monthly enrollment numbers in the overall population and/or the subpopulation (when its pattern is different from global) can be specified by the user when the recruitment is NOT uniform using the pop-out buttons. Based on the overall sample size and subpopulation percentage, the availability of the participants for monthly recruitment is provided for timely logic checks. The cell denoting available participants is in red color when the number is not equal to 0. The starting point of the subpopulation enrollment can be specified in a monthly manner.

The result table for TTE endpoint has more columns than other types of endpoints including two types of consistency probabilities (logHR and risk reduction) in conditional and unconditional way, time to target number of events in month, median durations of global and subpopulation in month.

### Non-Inferiority Design

For continuous and binary endpoints in NI designs which test the null hypothesis of treatment inferiority in difference by the NI margin (denoted as $\varDelta$ that is a positive number), the consistency probability [8] can be calculated as:

- Unconditional: $\:prob(\frac{{\widehat{\theta\:}}_{R}-\varDelta\:}{{\widehat{\theta\:}}_{All}-\varDelta\:}>\pi\:)$ for higher means worse setting,
  and $\:prob\left(\frac{{\widehat{\theta\:}}_{R}+\varDelta\:}{{\widehat{\theta\:}}_{All}+\varDelta\:}>\pi\:\right)$ for higher means better setting;
- Conditional: $$\:prob\left(\begin{array}{l}\frac{{\widehat{\theta\:}}_{R}-\varDelta\:}{{\widehat{\theta\:}}_{All}-\varDelta\:}>\pi\:|{\widehat{\theta\:}}_{All}\:is\\\:statistically\:significant\end{array}\right)$$
  for higher means worse setting,
$$\text{and}\:prob\left(\begin{array}{l}\frac{{\widehat{\theta\:}}_{R}+\varDelta\:}{{\widehat{\theta\:}}_{All}+\varDelta\:}>\pi\:|{\widehat{\theta\:}}_{All}\:is\\\:statistically\:significant\end{array}\right)$$
  for higher means better setting.

For TTE endpoint in NI designs, similar as in superiority designs, there are two ways of consistency calculation involving the NI margin:

1. Log HR: $$\:\frac{\text{log}\left({\widehat{HR}}_{R}\right)-\text{l}\text{o}\text{g}(\varDelta\:)}{\text{l}\text{o}\text{g}\left({\widehat{HR}}_{All}\right)-\text{l}\text{o}\text{g}(\varDelta\:)}>\pi\:$$

(2) Risk reduction: $$\:\frac{1-{\widehat{HR}}_{R}/\varDelta\:}{1-{\widehat{HR}}_{All}/\varDelta\:}>\pi\:$$

#### Illustration of NI Design

**Fig. 6:**
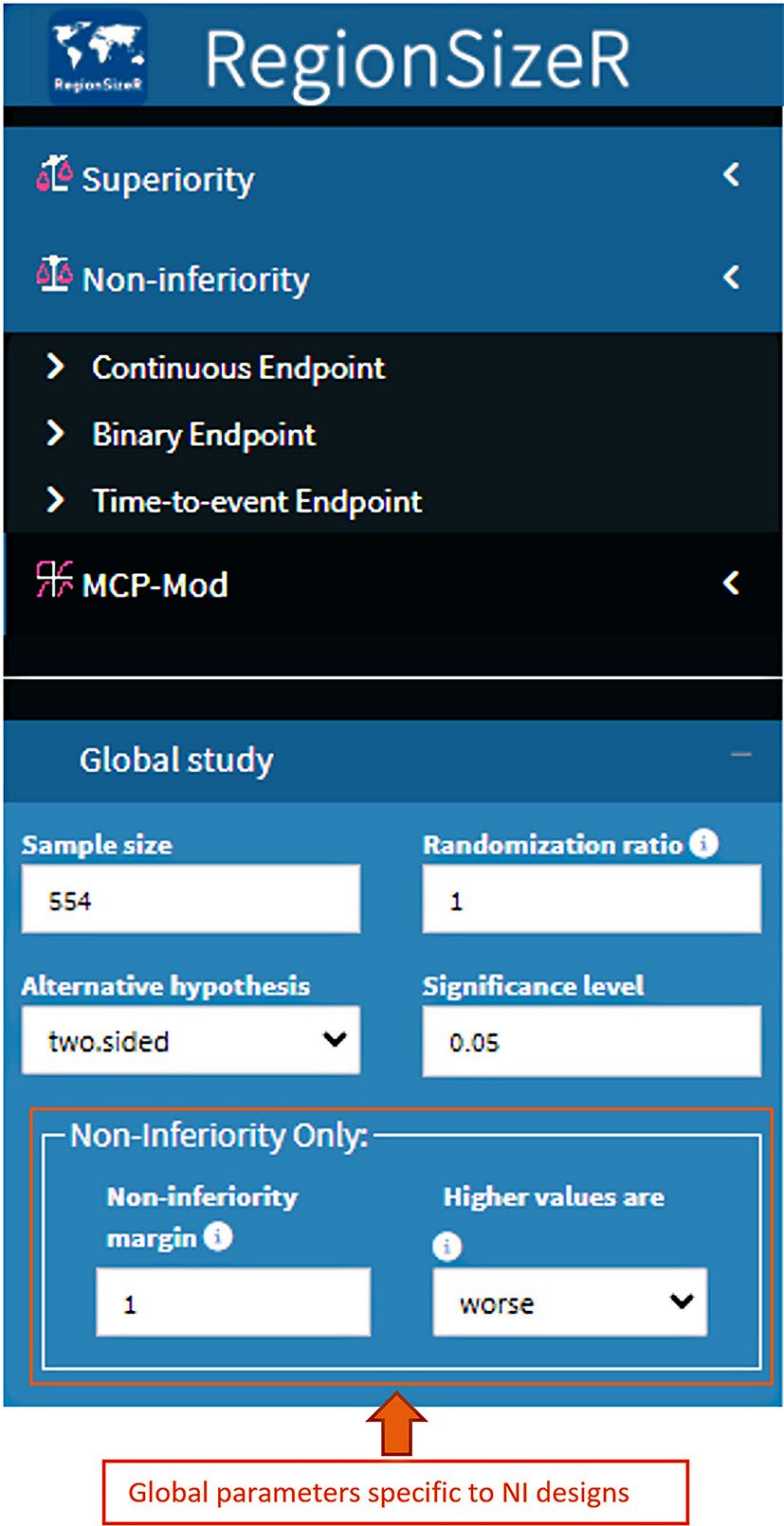
Screenshot of the parameters specific to NI design. Two additional parameters including the NI margin and treatment effect direction can be entered in the global parameter I panel

As shown in Fig. 6, the global parameters (part I) add two more parameters for NI design compared to the superiority design including: the NI margin and higher values are better/worse. With these two parameters used in the global study, users can evaluate the consistency based on the formula listed above according to the type of the endpoint. All the other parameters and the result tables remain similar as for the superiority design.

### MCP-Mod Design

For the MCP-Mod design, the app evaluates the treatment consistency based on two key assumptions:

- The proof-of-concept (PoC) is examined using the overall population and the dose-response model chosen for the overall population is ALSO used for the region of interest;
- All calculations are performed under the condition that the dose-response signal is significant for the overall population. That is being said, only the conditional probability is calculated for MCP-Mod design.

Denote $\:{\{c}_{i}^{*},\:i=1,\:\dots\:,d\}$ as the coefficients of the contrast corresponding to the best dose-response model chosen from the candidate set to establish PoC, $\:d$ is the number of dose levels including placebo of the dose-finding study, *μ*_*m*_ is the expected mean response of the assumed true model m (where m = 1,…, M that is the total number of candidate models considered for study sample size determination), *n*_*R*_ is the number of participants in the region of interest, and $\bar Y_i$ and $\bar Y_i^R$ are the mean responses of the simulated overall and region data at dose $\:i$. For continuous and binary endpoints in the MCP-Mod design, the conditional consistency probability can be calculated by the local consistency in contrast statistic [9] defined as:

$$\:Prob\left(\begin{aligned}\frac{{\sum\:}_{i=1}^{d}{c}_{i}^{*}{\stackrel{-}{Y}}_{i}^{R}\left({n}_{R}\right)}{{\sum\:}_{i=1}^{d}{c}_{i}^{*}{\stackrel{-}{Y}}_{i}}>\pi\:|\mu\:=&{\mu\:}_{m},\:global\:statistically\\ &\:significant\:signal\end{aligned}\right)$$

In this formula, please note that instead of the simulated means at dose levels for continuous type of endpoint, the logit values derived from the simulated response rates are used for binary type of endpoint, following the same approach as in ‘DoseFinding’ package [10]. Consequently, the interpretation of the consistency probability for binary endpoint is based on the logit scale rather than the response rate. When a clinically relevant treatment effect size Δ is specified besides PoC, the median value of minimum effective dose (MED) can be predicted using the chosen best dose-response model based on the simulated data in the overall population. Together with overall power, these two columns of result can be used to cross check with overall sample size calculation.

Furthermore, for the criteria of global statistically significant dose-response signal, there are three types of option provided in the app to match with study sample size determination:

1. For PoC only, i.e. there is at least one statistically significant dose-response signal in the candidate model set based on the multiple contrast test;
2. PoC and the estimated treatment effect size > Δ (when available, or < - Δ depending on the effect direction) in at least one active dose;
3. PoC, estimated treatment effect size > Δ (or < - Δ) in at least one active dose, and modeling of MED is within the designed dose range.

#### Illustration of Contrast Based Consistency

**Fig. 7:**
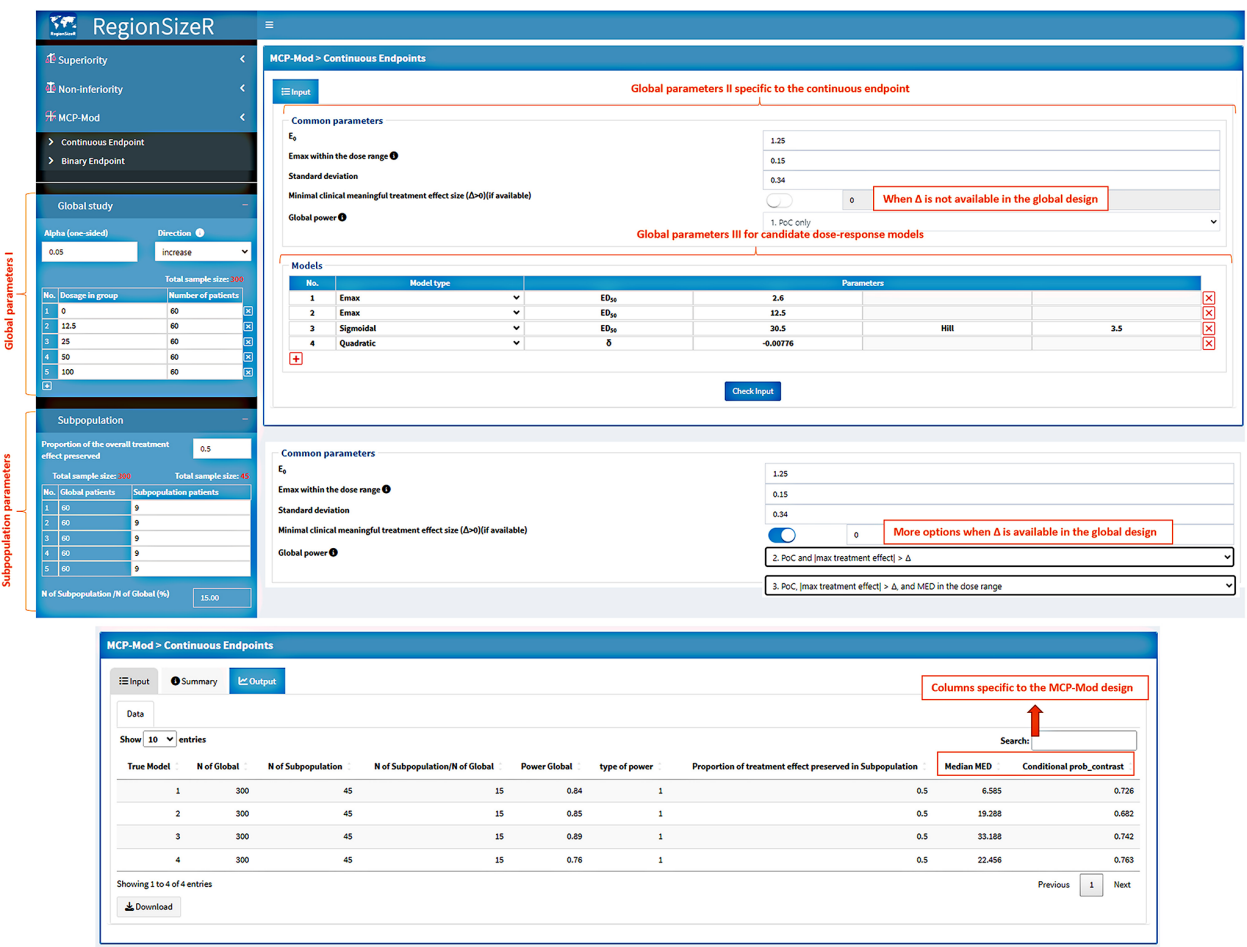
Screenshot of the parameters and result table specific to MCP-Mod design. The number of participants at each dose level can be specified in global study and subpopulation panels. Other global parameters including candidate dose-response models are illustrated using continuous endpoint as an example. Depending on the availability of Δ, three types of overall power can be selected to match with study design and median MED shall be calculated in the result table

We use the continuous endpoint as an example to illustrate the MCP-Mod design as shown in Fig. 7. In this example, there are 5 dose levels from 0 (i.e. the placebo arm) to 100 unit with 60 participants in each level. The increase in the response variable is beneficial with E_0_ = 1.25 and E_max_=0.15 within the dose range over placebo (standard deviation = 0.34). For the subpopulation parameters, to preserve at least ½ of the global treatment effect, the consistency is evaluated when 15% of the overall participants coming from the region of interest (9 participants in each dose level). When there is no minimal clinical meaningful treatment effect size (Δ) available in the global study design, only PoC option (type 1 power) can be applied for the overall power in the simulation. When Δ is available in the global study used for overall power estimation or MED calculation, the other two types of overall power (type 2 and 3) can be applied in the simulation accordingly.

In the Models panel located in the lower right part of the app, all candidate models used for the global MCP-Mod design are specified with models of the same type listed neighboring each other. In the illustrated example, there are 4 candidate models where the first two models both are Emax model with different ED_50_ parameters (2.6 and 12.5). Another Sigmoidal model and Quadratic model are listed afterward as the 3rd and 4th candidate model. All the input is summaried in the summary tab incorperating logic checks based on the natural relationship among parameters. Finally, the result table including the median MED across simulation runs (if Δ is provided) and the constrast based conditional consistency probability is presented in the Output tab.

For the binary endpoint, the usage of the app is similar as for the continuous endpoint but based on the response rates when defining E_0_ and other related parameters. Accordingly, the contrast based conditional consistency probability defined above is calculated in the logit scale.

## Discussion and Conclusion

In this paper, we introduce a novel app *RegionSizeR*, designed for efficient and harmonized way of regional sample size planning in multi-regional clinical trials. The app is intended for use during the planning stage of an MRCT to assist with regional sample size allocation and empowers users to perform consistency predictions based on simulated data by means of a user-friendly interface. The app accommodates region-specific considerations via the parameter setting panel which can be downloaded/uploaded by the users as needed.

Hypothetical parameters representing commonly seen designs in late phase trials are utilized for illustrating the functions of the app. Validation by independent programming and logic checks ensure the results provided by the app are accurate and reliable, with a discrepancy allowance of < = 1% except in rare cases. During the test of region partially included in global study for TTE endpoint in a NI design, the consistency probabilities may vary within a relatively wide range (± 5%), particularly in extreme cases where the overall power is quite low (for instance, lower than 70% based our simulated testing) or when the number of simulation runs is insufficient. In the test for an MCP-Mod design with binary endpoint, the consistency probabilities may vary by up to 2% when the regional sample size at each dose level is relatively small (e.g., fewer than 5 participants per dose level). Consequently, we recommend that users increase the number of simulation runs for more robust results or employ additional tools for further confirmation. Additionally, despite our validation efforts, it is not guaranteed that all scenarios have been tested. Therefore, we recommend that users perform their own validation based on specific circumstances before using it for any official purposes.

The *RegionSizeR* app can serve as a starting point for discussions on regional sample size allocation plans, adhering to the preservation of treatment effect method in ICH E17; However, it is not designed to replace the sample size determination for MRCTs. Instead, it can be utilized to replicate and cross-check the study power which is the foundation for regional consistency evaluations.

Moreover, this app can be generalized and applied to plan the subgroup sizes that are not limited to regions or countries within a study, which is a particularly useful feature when a certain level of consistency is expected in the results among specific subgroups.

Of course, there are also limitations of the app. For instance, only pair-wise comparisons can be applied in the app, hence if the MRCT is a single arm design, the regional sample size planning cannot be finished using this app. Furthermore, for designs using multiple comparisons, users must perform multiplicity control procedures prior to applying the app. Since the app can only handle three types of endpoints (continuous, binary, and TTE) that are commonly used as primary efficacy endpoints in late-phase confirmatory trials, other types of endpoints, such as rank-based endpoints, are not included at this stage of development. Additionally, the running speed of this simulation-based Shiny app is a limitation, especially when the number of iterations exceeds 1,000.

In conclusion, the deployment of the app within our company has garnered many thumbs up. The app has been well acknowledged by regional project teams and statisticians for its potential to facilitate efficient and harmonized planning. The current options and features of the app were implemented through a feedback-based optimization mode, in close collaboration with statistical analysts in the working group. The app can be obtained from https://github.com/rsr-ss/RegionSizeR. Should there be a need for additional features, users with an R programming background have the opportunity to customize the app to meet their specific requirements.

## Electronic supplementary material

Below is the link to the electronic supplementary material.

Supplementary Material 1

## Data Availability

No datasets were generated or analysed during the current study.
