# Supplementary material for: RegionSizeR– A Novel App for Regional Sample Size Planning in MRCTs
Source: Ther Innov Regul Sci. 2024 Aug 8;58(6):1071–9. doi: 10.1007/s43441-024-00679-6 (PMC11530476; doi:10.1007/s43441-024-00679-6)
Supplement: Supplementary file 1 — Supplementary Material 1 [file 43441_2024_679_MOESM1_ESM.docx]

# Supplementary Material

**Supplementary Table 1** an example of the validation plan used for binary endpoint in the superiority design.

| **Parameter** | **Test setting** |
| --- | --- |
| Threshold for preservation of treatment effect | 1/2, 1/3, and 0 |
| Sample size | 100, 300, and 700 |
| Response proportions (treatment vs placebo) | 0.6 vs 0.3 for sample size of 100  0.5 vs 0.3 for sample size of 300  0.4 vs 0.3 for sample size of 700  (So that the overall powers would be at least above 80%) |
| Treatment allocation in subpopulation | Balanced (1:1) / unbalanced (2:1 for size of 100 and 300; or 3:1 for size of 700) |
| Subpopulation percentage | 10%, 15%, and 20% |
| Relationship between subpopulation and overall | Completely included, completely excluded, and partially included (20%, 50%, and 80%) |
